# A role for *VAX2* in correct retinal function revealed by a novel genomic deletion at 2p13.3 causing distal Renal Tubular Acidosis: case report

**DOI:** 10.1186/s12881-015-0182-1

**Published:** 2015-06-13

**Authors:** Elizabeth E. Norgett, Anthony Yii, Katherine G. Blake-Palmer, Mostafa Sharifian, Louise E. Allen, Abdolhamid Najafi, Ariana Kariminejad, Fiona E. Karet Frankl

**Affiliations:** Departments of Medical Genetics, Renal Medicine and Ophthalmology, University of Cambridge, Cambridge, UK; Pediatric Nephrology Research Center, Pediatric Infections Research Centre (PIRC), Shaheed Beheshti University of Medical Sciences, Tehran, Iran; Azad University Medical Branch, Tehran, Iran; Kariminejad-Najmabadi Pathology and Genetics Centre, Tehran, Iran; Cambridge Institute for Medical Research, Box 139, Cambridge Biomedical Campus, Cambridge, CB2 0XY UK

**Keywords:** dRTA, H^+^-ATPase, *ATP6V1B1*, *VAX2*, Retina

## Abstract

**Background:**

Distal Renal Tubular Acidosis is a disorder of acid-base regulation caused by functional failure of α-intercalated cells in the distal nephron. The recessive form of the disease (which is usually associated with sensorineural deafness) is attributable to mutations in *ATP6V1B1* or *ATP6V0A4*, which encode the tissue-restricted B1 and a4 subunits of the renal apical H^+^-ATPase. *ATP6V1B1* lies adjacent to the gene encoding the homeobox domain protein VAX2, at 2p13.3. To date, no human phenotype has been associated with *VAX2* mutations.

**Case presentation:**

The male Caucasian proband, born of a first cousin marriage, presented at 2 months with failure to thrive, vomiting and poor urine output. No anatomical problems were identified, but investigation revealed hyperchloremic metabolic acidosis with inappropriately alkaline urine and bilateral nephrocalcinosis. Distal Renal Tubular Acidosis was diagnosed and audiometry confirmed hearing loss at 2 years. *ATP6V0A4* was excluded from genetic causation by intragenic SNP linkage analysis, but *ATP6V1B1* completely failed to PCR-amplify in the patient, suggesting a genomic deletion. Successful amplification of DNA flanking *ATP6V1B1* facilitated systematic chromosome walking to ascertain that the proband harbored a homozygous deletion at 2p13.3 encompassing all of *ATP6V1B1* and part of *VAX2*; gene dosage was halved in the parents. This results in the complete deletion of *ATP6V1B1* and disruption of the *VAX2* open reading frame. Later ocular examinations revealed bilateral rod / cone photoreceptor dystrophy and mild optic atrophy. Similar changes were not detected in an adult harbouring a disruptive mutation in *ATP6V1B1.*

**Conclusions:**

The genomic deletion reported here is firstly, the only reported example of a whole gene deletion to underlie Distal Renal Tubular Acidosis, where the clinical phenotype is indistinguishable from that of other patients with *ATP6V1B1* mutations; secondly, this is the first reported example of a human *VAX2* mutation and associated ocular phenotype, supporting speculation in the literature that *VAX2* is important for correct retinal functioning.

## Background

Most physiological functions and the ultimate survival of all multicellular organisms depend on the maintenance of constant extracellular pH (close to 7.4 in man) and appropriate adaptations to change. The normal human omnivorous diet creates a net acid load, which requires excretion. The kidney plays a vital part in pH regulation by extruding acid into nascent urine, with concomitant reabsorption of bicarbonate. The two main sites for acid-base regulation along the nephron (the functional unit of the kidney) are the proximal tubule, where major bicarbonate reabsorption takes place, and the distal nephron where fine regulation is achieved by specialized acid-handling epithelial cells, α-intercalated cells (α-ICs). At this site, the net effect in humans is acid secretion [1].

Alpha-ICs are polarized such that H^+^ secretion occurs across their apical plasma membrane and into the urine by specialized multi-subunit proton pumps (H^+^-ATPases). H^+^-ATPases are also vital for acidification of intracellular compartments such as endosomes and lysosomes. They consist of two domains, a catalytic V_1_ domain responsible for ATP hydrolysis, and a membrane-anchored V_0_ domain that uses the energy thus generated to drive H^+^ transport. Each domain comprises a number of separate components such that together, the pump contains a total of at least 13 different subunits, all separately encoded [2].

On the basolateral side of α-ICs, the anion exchanger AE1 (encoded by *SLC4A1*) acts as a 1:1 chloride/bicarbonate exchanger, where it reabsorbs bicarbonate. How apical H^+^-ATPases and AE1 are functionally coupled is not understood [1].

Failure of acid-base regulation in α-ICs results in distal Renal Tubular Acidosis (dRTA; MIM#602722 and 267300). This disease is characterized by sometimes-severe hyperchloremic metabolic acidosis with an inappropriately alkaline urine (pH >5.5), usually accompanied by hypokalemia, hypercalciuria, renal tract calcification and in untreated cases, rickets/osteomalacia. It can occur secondarily, particularly in autoimmune diseases such as Sjogren’s syndrome, or can be inherited as either an autosomal dominant or recessive disorder. Dominant dRTA is caused by mutations in *SLC4A1* [3, 4]. We and others have previously shown that mutations in *ATP6V1B1* and *ATP6V0A4*, which encode subunits B1 and a4 of the H^+^-ATPase respectively, are causative of recessive dRTA (rdRTA). These are associated with progressive sensorineural hearing loss (SNHL) [5–7], which is presumed due to endolymphatic pH abnormalities [5–7]. a4 and B1 are tissue-restricted forms of the a- and B subunits respectively, who replace the ubiquitously expressed a1 and B2 forms found on intracellular membranes. Their expression is largely restricted to the apical surface of the α-IC, male reproductive tract and inner ear.

*ATP6V1B1* maps to 2p13.3, adjacent to *VAX2*, which encodes a homeobox protein transcription factor that is almost exclusively expressed in the ventral portion of the retina during development. To date, no human mutations have been found in *VAX2*, but GWAS meta-analysis has suggested it as a candidate to underlie astigmatism [8]. *Vax2-*null mice show disruptions to the establishment of a dorsoventral axis of the neural retina, a key step in eye development, and abnormal outgrowth of ventral retinal ganglion cells [9, 10]. Transcriptome analysis in these mice suggests that lack of *Vax2* expression adversely affects the distribution of retinoic acid which itself plays a major part in correct patterning of the eye during development. Importantly, retinoic acid is also shown to be important in maintenance of appropriate gene expression in the photoreceptor cells of the mature retina [11]. Some of the *Vax2*^*-/-*^ mice also display incomplete closure of the optic fissure and coloboma (with variable penetrance) and there is speculation in the literature that *VAX2* might be responsible for a similar human phenotype [9, 12].

*VAX2* shares 100 % identity in the homeobox domain with *VAX1*, which is strongly expressed both during early development in the neural ridge, and later in the rostro-medial olfactory placode, optic nerve and disc and anterior ventral forebrain. VAX1 is known to be essential both for corpus callosum development and correct axon guidance in the optic nerve and forebrain [13]. A single case has been reported wherein a mutation in *VAX1* (R152S) was found in a patient with microphthalmia, optic nerve hypoplasia, cleft lip/palate and corpus callosum agenesis, a phenotype similar to that found in the *Vax1* null mouse [13, 14]. *VAX2* was also screened in an additional 70 patients with anophthalmia/microphthalmia but no mutations were found [14]. To date therefore, the single *VAX1* case represents the only reported mutation in either human VAX gene.

Here, we describe a genomic deletion that causes both dRTA (due to the complete absence of *ATP6V1B1*) and a novel deleterious eye phenotype (due to disruption of *VAX2*). This deletion represents a novel mechanism of disease for dRTA and underpins the importance of *VAX2* in maintenance of retinal integrity in man.

## Case presentation

### Clinical presentation

The consanguineous Caucasian kindred under study was referred via pediatric nephrology services in Tehran, Iran. The male patient, born to first cousin parents, presented at 2 months of age with failure to thrive and vomiting, and was described as having ‘difficulty in urination’. On examination there were no anatomical problems, but hyperchloremic metabolic acidosis with inappropriately alkaline urine were discovered on biochemical assessment (blood pH 7.01, HCO_3_ 4mmol/l, urine pH > 6). Ultrasonography revealed bilateral nephrocalcinosis. Hearing impairment was suspected at two years old and confirmed by audiometry. Visual difficulties were not reported, and both parents and a sibling were clinically normal.

### Genetic investigation of dRTA

**Fig. 1:**
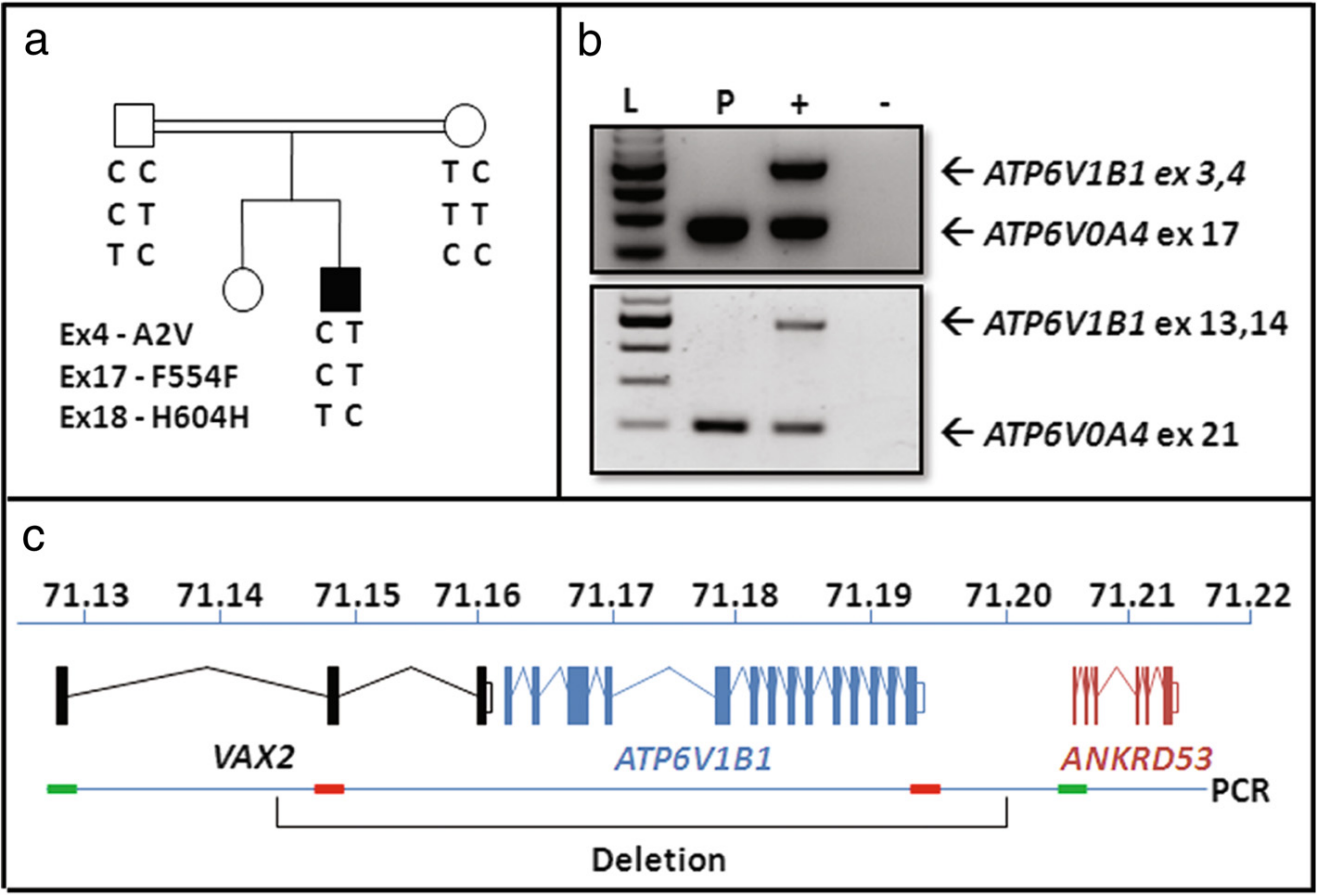
Evidence that the deletion of *ATP6V1B1* underlies dRTA in this family (**a**) *ATP6V0A4* was excluded by heterozygosity of SNPs in the patient in exons 4, 17 and 18. **b** Failure of PCR to amplify exons 3–4 and 13–14 of *ATP6V1B1* in the patient (P) suggested a large deletion compared to positive control (+). **c** Successful PCR amplification in the patient for exon 1 of *VAX2* () and exon 1 of *ANKRD53* (), but not exon 2 of *VAX2* () and exon 21 of *ATP6V1B1* () define regions on either side of *ATP6V1B1* for the deletion breakpoints on chromosome 2. L = 100bp ladder

**Fig. 2:**
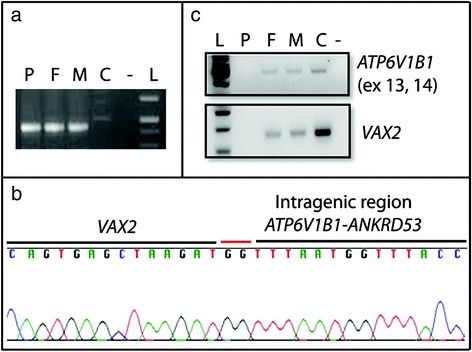
Characterization and gene dosage of the genomic deletion at 2p13.3 (**a**) Successful PCR amplification across the junction breakpoint in the patient (P) and his parents (F, M) but not in a control individual with normal genomic DNA (C). **b** Sequencing revealed the insertion of 2 nucleotides (GG) at the site of the deletion. **c** Semi-quantitative PCR confirmed that gene dosage of *ATP6V1B1* and exons 2 and 3 of *VAX2* is reduced in the parents (F, M) compared to the control (C)

Linkage analysis using previously described intragenic SNPs within both *ATP6V0A4* and *ATP6V1B1* excluded linkage to *ATP6V0A4* in this family [7] (Fig. 1a). In contrast, none of the intragenic SNPs in *ATP6V1B1* would PCR-amplify in the patient. Multiplex PCR amplification of both genes confirmed the integrity of the DNA template, suggesting a genomic deletion (Fig. 1b). To investigate the extent of the deletion, genes flanking *ATP6V1B1* were also subjected to PCR in the proband and an unrelated unaffected individual. *VAX2* lies immediately 5′ of *ATP6V1B1*, and *ANKRD53* is 3′ (Fig. 1c). In the patient, successful PCR amplification was achieved for exon 1 but not exons 2 or 3 of *VAX2*, indicating that the deletion breakpoint lay in intron 1. All exons of *ANKRD53* were successfully amplified. Further PCR was performed in the patient and an unrelated unaffected individual to ‘walk’ systematically inwards until a segment was amplified from control but not patient DNA. Reactions were performed using QIAGEN’s multiplex PCR kit in a total reaction volume of 15 μl containing 50 ng DNA. Finally, the outermost primers of these pairs (*VAX2*, intron 1 and between *ATP6V1B1* and *ANKRD53*) were used in combination to amplify across the presumed deleted region in the proband (Fig. 2a). Primer sequences are available on request. Sequencing of this product was performed and analyzed by BLAT [15]. The first 225 bp exactly matched sequence in intron 1 of *VAX2*. This was followed by GG, and the remainder of the product exactly matched sequence from the region between *ATP6V1B1* and *ANKRD53* (Fig. 2b)*.* This confirmed that the deleted region was 56.8 Kb and contained 2 of the 3 coding exons of *VAX2* and the known dRTA gene.

Gene dosage in family members was investigated by semi-quantitative PCR, using primers to amplify exons 13–14 of *ATP6V1B1* and exon 3 of *VAX2* from genomic DNA of the patient, both parents and an unrelated unaffected individual. This confirmed that both parents were heterozygous for the deletion (Fig. 2c).

### Investigation of a possible ocular phenotype due to loss of *VAX2*

**Fig. 3:**
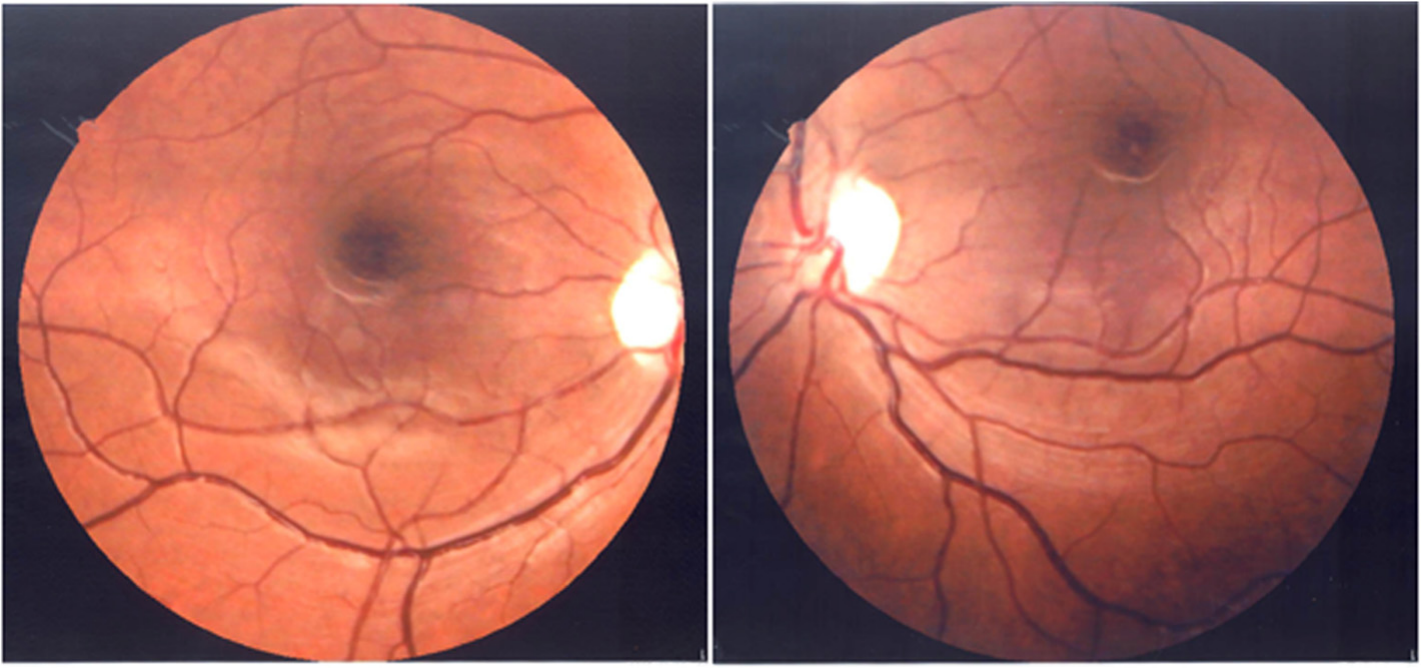
Fundus photographs of the right eye (left image) and left eye (right image). Abnormal pigmentation around the macula and mild bilateral optic atrophy can be seen.

No visual problems were initially reported in this patient, now aged 13. However, recent retrospective ophthalmological examination revealed subtle abnormalities. Corrected visual acuity was 18/20 in each eye. Refraction showed [OD +1.50 DS -0.75 DC at 12^0^] and [OS +1.0 DS -0.75 DC at 160^0^]. Morphology of the external eye was normal bilaterally, with normal ocular motility and anterior segments. Fundal examination revealed bilateral pigmentary changes at the macula and retinal periphery consistent with a retinal dystrophy, and bilateral optic atrophy (Fig. 3). Electroretinography demonstrated reduced amplitude a-waves in keeping with a photoreceptor dystrophy. Visual evoked potential amplitude was mildly reduced, likely secondary to ganglion cell loss from the retinal dystrophy.

To confirm that the phenotype seen in this patient was not attributable to B1 deficiency, we arranged for detailed retinal examination of an unrelated adult (32 year old) dRTA patient with an underlying homozygous B1 mutation (c.687 + 1G > T; a previously reported [5] essential splice site mutation at the exon 7/intron 7 boundary). This revealed normal retinal function with no cellular changes (data not shown), which supports the conclusion that *VAX2* deficiency in the patient described here is responsible for the eye phenotype.

## Conclusions

This is the first time that a whole gene deletion has been found to underlie dRTA. The clinical phenotype in this patient was indistinguishable from cases caused by nonsense or missense mutations in *ATP6B1V1*. However, on finding the deletion of two of the three coding exons of *VAX2*, a post-hoc ocular examination revealed an undiagnosed eye phenotype. The patient did not manifest coloboma but this is not particularly surprising as in *Vax2*^*-/-*^ mice this abnormality showed variable penetrance [9, 10]. However, the patient described here was found to have electrophysiological evidence of photoreceptor degeneration. Additionally, there was optic atrophy similar to *Vax2*^*-/-*^ mice which may be primary or secondary to the retinal dystrophy.

A possible mechanism for the abnormality is disruption to retinoic acid disposition in the retina, for which VAX2 is necessary, and which is important for photoreceptor function [11]. Both in this patient and in the *Vax2*-null mouse, the phenotype is mild compared to that associated both with a previously reported human *VAX1* mutation and a targeted murine *Vax1* deletion, where coloboma was fully penetrant and moderately severe [13, 14]. The mild VAX2-null phenotype is likely due to compensation by *VAX1* with which *VAX2* has been shown to work in concert to ventralize the eye. *Vax1/Vax2* double mutant mice have a fully penetrant, severe phenotype where the optic nerve is transformed into differentiated retina. This is caused by loss of repression of the Pax6 transcription factor, which promotes a dorsal phenotype/retinal differentiation [16].

The normal retinal function we report in an unrelated, adult dRTA patient with a homozygous B1 splice site mutation excludes the possibility that the eye phenotype seen in the case described here is attributable to a failure of the H^+^-ATPase. We and others have previously demonstrated expression of the tissue-restricted *ATP6V0A4* and *ATP6V1B1* in the eye [17–19] where it is suggested that the proton pump contributes to intraocular fluid homeostasis. Additionally, the role of the a3 subunit of the H^+^-ATPase in correct ocular function is well documented; mutations cause severe autosomal recessive osteopetrosis often associated with impaired vision [20] but here, loss of sight is attributed to compression of the optic nerve caused by failure of bone resorption by osteoclasts [18].

This study is important in terms of clinical and research findings as it has confirmed that VAX2 plays an important role in maintenance of the human neural retina. In order to further understand the pathogenesis, more patients with *VAX2* mutations will need to be investigated. Although presentation of these patients has not to date been forthcoming, they may now be easier to identify as we have some *a priori* knowledge of how the phenotype might present.

## Consent

Written informed consent was obtained from the parents of the index case (Cambridge Local Research Ethics Committee 99/078). A copy of the written consent is available for review by the Editor of this journal.
